# The X-ray structure of human calbindin-D28K: an improved model

**DOI:** 10.1107/S2059798318011610

**Published:** 2018-10-02

**Authors:** James W. Noble, Rehab Almalki, S. Mark Roe, Armin Wagner, Ramona Duman, John R. Atack

**Affiliations:** a Sussex Drug Discovery Centre, University Of Sussex, Falmer, Brighton BN1 9QG, England; b School Of Life Sciences, University Of Sussex, Falmer, Brighton BN1 9QG, England; c Diamond Light Source, Harwell Science and Innovation Campus, Chilton, Didcot OX11 0DE, England; d Medicines Discovery Institute, Cardiff University, Cardiff CF10 3AT, Wales

**Keywords:** calbindin, calcium, calbindin-D28K, long wavelength, calcium SAD

## Abstract

Calbindin-D28K is a widely expressed calcium-buffering cytoplasmic protein that is involved in many physiological processes. It has been shown to interact with other proteins, suggesting a role as a calcium sensor. Many of the targets of calbindin-D28K are of therapeutic interest: for example, inositol monophos­phatase, the putative target of lithium therapy in bipolar disorder. Presented here is the first crystal structure of human calbindin-D28K. There are significant deviations in the tertiary structure when compared with the NMR structure of rat calbindin-D28K (PDB entry 2g9b), despite 98% sequence identity. Small-angle X-ray scattering (SAXS) indicates that the crystal structure better predicts the properties of calbindin-D28K in solution compared with the NMR structure. Here, the first direct visualization of the calcium-binding properties of calbindin-D28K is presented. Four of the six EF-hands that make up the secondary structure of the protein contain a calcium-binding site. Two distinct conformations of the N-terminal EF-hand calcium-binding site were identified using long-wavelength calcium single-wavelength anomalous dispersion (SAD). This flexible region has previously been recognized as a protein–protein interaction interface. SAXS data collected in both the presence and absence of calcium indicate that there are no large structural differences in the globular structure of calbindin-D28K between the calcium-loaded and unloaded proteins.

## Abstract 2

The X-ray structure of human calbindin-D28K, a calcium-buffering protein that is highly expressed in the central nervous system, is reported.

## 1. Introduction   

Calbindin-D28K is a major calcium-buffering cytoplasmic protein that is expressed at particularly high levels in the central nervous system (CNS) and absorptive epithelium (gut and kidney; Schmidt, 2012 ▸). Calcium signalling is tightly regulated and is involved in a myriad of physiological processes, and consequently calcium deregulation is a key factor in the pathogenesis of many diseases, such as Alzheimer’s disease (Bojarski *et al.*, 2008 ▸; Kook *et al.*, 2014 ▸). Calbindin-D28K was first identified in the intestine, colon, kidney and uterus of *Gallus gallus domesticus* (chicken; Wasserman *et al.*, 1969 ▸), where it is involved in the transcellular movement of calcium across the absorptive epithelium, as found in the distal convoluted tubules of the kidney (Lambers *et al.*, 2006 ▸). Calbindin-D28K is also highly expressed in the CNS, where it contributes up to 1.5% of the total soluble protein (Christakos *et al.*, 1989 ▸; Berggård, Szczepankiewicz *et al.*, 2002 ▸). In chicken kidney cells the expression of calbindin-D28K is vitamin D dependent, and this is true for other absorptive cells (Clemens *et al.*, 1989 ▸); however, it is not the case in the CNS (Arnold & Heintz, 1997 ▸). Calbindin-D28K has been reported to regulate the depolarization-stimulated release of insulin from pancreatic β cells through the regulation of the cytoplasmic calcium concentration (Sooy *et al.*, 1999 ▸). It is well documented in the literature that calbindin-D28K has neuroprotective properties in the CNS (Yenari *et al.*, 2001 ▸; Yuan *et al.*, 2013 ▸; Sun *et al.*, 2011 ▸), and it has recently been demonstrated that its depletion in an Alzheimer’s disease mouse model accelerates neuronal loss, apoptosis and mitochondrial dysfunction (Kook *et al.*, 2014 ▸).

It has been shown that calbindin-D28K interacts with a variety of proteins of therapeutic interest (Schmidt, 2012 ▸). Calbindin-D28K binds and increases the catalytic activity of inositol monophosphatase (IMPase), the putative target of lithium therapy in bipolar disorder (Berggård, Szczepankiewicz *et al.*, 2002 ▸). IMPase has a key role in the homeostasis of the IP3 signalling cascade by replenishing free *myo*-inositol, and inhibition of this enzyme by lithium gave rise to the inositol-depletion hypothesis for the efficacy of lithium in the treatment of bipolar disorder (Harwood, 2005 ▸). More recently, IMPase inhibition has been shown to increase autophagy and the clearance of peptides involved in the pathogenesis of both Huntington’s and Parkinson’s diseases (Sarkar *et al.*, 2005 ▸), indicating that inhibition of the calbindin–IMPase interaction may be of therapeutic benefit in these diseases. The calbindin–IMPase interaction has been modelled *in silico* and the information has been used to develop a series of novel peptide inhibitors (Levi *et al.*, 2013 ▸). Calbindin-D28K also exhibits anti-apoptotic activity by binding to and inhibiting caspase-3 (Bobay *et al.*, 2012 ▸; Bellido *et al.*, 2000 ▸). Understanding the structural basis of these interactions would offer possible new therapeutic approaches to tackling these diseases.

Calbindin-D28K belongs to a superfamily of calcium-binding proteins that includes calmodulin and troponin C. All of these proteins have a high α-helical content and share EF-hand structures that constitute the calcium-binding domains. Calbindin-D28K has a primary structure of 261 amino acids (molecular mass of ∼30 kDa) and is encoded by the *CALB1* gene. In 2006 an NMR structure of the calcium-loaded *Rattus norvegicus* calbindin-D28K protein revealed the tertiary fold of the protein for the first time: calbindin-D28K is mainly α-helical and the α-helices make up six EF-hand motifs that are held together in a single globular fold *via* hydrophobic interactions (Kojetin *et al.*, 2006 ▸). EF-hand motifs have a helix–loop–helix topology and are a common structural characteristic in calcium-binding proteins. As in most calcium-binding proteins, the calcium ion is coordinated in the loop region between the two helices of the EF-hand; in calbindin-D28K a calcium ion is coordinated in four of the six EF-hand motifs. EF-hands 1, 3, 4 and 5 bind calcium with high affinity (Åkerfeldt *et al.*, 1996 ▸; Venters *et al.*, 2003 ▸). It has been shown that calbindin-D28K undergoes structural changes upon calcium binding indicative of a calcium-sensing protein (Berggård, Miron *et al.*, 2002 ▸). However, both the apo and the calcium-loaded protein have exposed hydrophobic residues on the surface, indicating that calbindin-D28K interacts with other proteins in both states (Berggård *et al.*, 2000 ▸). The high-resolution crystal structure of human calbindin-D28K reported here confirms these secondary-structural findings and also reveals some significant differences when compared with the NMR structure of rat calbindin-D28K. The human calbindin-D28K structure presented here allows the first direct visualization of calcium ions bound by the protein.

## 2. Methods   

### 2.1. Cloning and expression   

The *Escherichia coli* codon-optimized human *CALB1* gene was synthesized and cloned into pET-15b using NcoI and BamHI restriction sites (GenScript). A polyhistidine tag followed by a rhinovirus (HRV) 3C protease cleavage sequence was included in the synthetic construct directly 5′ to the calbindin-D28K start codon. The recombinant plasmid was then transformed into *E. coli* Rosetta 2 (DE3) cells (Novagen) and plated on LB agar containing 50 µg ml^−1^ ampicillin. A 100 ml preculture was grown in LB broth (50 µg ml^−1^ ampicillin) overnight at 310 K with shaking at 200 rev min^−1^. 10 ml of the preculture was used to inoculate the main 1 l cultures, which were grown at 310 K with shaking at 200 rev min^−1^ to an OD_600_ of ∼0.6. The cultures were incubated on ice for 2 h before IPTG induction (0.4 m*M*) and incubation overnight at 298 K with shaking at 180 rev min^−1^.

### 2.2. Purification   

The cells were harvested and spun at ∼9500*g* for 20 min, and the pellet was resuspended in sonication buffer [150 m*M* NaCl, 1 m*M* MgCl_2_, 20 m*M* Tris pH 8.0 and protease-inhibitor cocktail (cOmplete, EDTA-free, Roche)]. Cell lysis was achieved by sonication for 5 min with 5 s intervals and DNA was removed by the addition of a nonspecific endonuclease (Benzonase nuclease, Sigma) and incubation on ice for 10 min. The lysate was clarified at ∼30 000*g* for 20 min and the supernatant was retained. His-tagged recombinant protein was purified by cobalt-affinity chromatography (Talon metal-affinity resin); the clarified lysate supernatant was incubated with 10 ml binding buffer-equilibrated resin for 1 h at 277 K (20 m*M* Tris pH 8.0, 150 m*M* NaCl, 10 m*M* imidazole). The flowthough was removed and the resin was sequentially washed with seven column volumes of binding buffer. The bound proteins were then eluted with binding buffer containing 250 m*M* imidazole. The His tag was removed by incubation with HRV 3C protease overnight at 277 K. The cleaved protein was further purified by size-exclusion chromatography on a Superdex G75 column in 5 m*M* Tris pH 8.0, 1 m*M* CaCl_2_, yielding ∼25–30 mg calbindin-D28K per litre of culture.

### 2.3. Crystallization   

The calbindin-D28K crystals that initially yielded the calcium SAD phases were replicated from previously reported crystallization conditions (crystallization condition 1: ∼60 mg ml^−1^ protein in 1 m*M* CaCl_2_, 5 m*M* Tris pH 8.0 added in a 1:1 ratio to 0.5 *M* ammonium acetate, 0.1 *M* bis-Tris pH 6.5, 24% PEG 3350; Zhang *et al.*, 2008 ▸). The crystal structure reported here was obtained using the JCSG+ Screen (Newman *et al.*, 2005 ▸) from Molecular Dimensions [crystallization condition 2: 0.1 *M* potassium thiocyanate, 30%(*w*/*v*) PEG 2000 MME]. Recombinant calbindin-D28K was concentrated to ∼40 mg ml^−1^ in 1 m*M* CaCl_2_, 5 m*M* Tris pH 8.0. Crystallization was performed in MRC 2-well crystallization plates (Swissci) with a reservoir volume of 50 µl and a drop volume of 0.2 µl consisting of a 1:1 ratio of precipitant solution and protein solution. The crystallization plates were incubated at 293 K and calbindin-D28K crystals grew within a week.

### 2.4. Data collection and processing   

X-ray diffraction data were collected from calbindin-D28K crystals grown under condition 1 on the long-wavelength macromolecular crystallography beamline I23 at Diamond Light Source (DLS; Wagner *et al.*, 2016 ▸). The calcium SAD phasing experiment was performed at a wavelength of 3.02 Å, close to the Ca *K* absorption edge (λ = 3.07 Å). A total of 8000 images from two 400° sweeps were collected with a 0.1 s exposure time using the inverse-beam method. The data were processed with the *DIALS* and *AIMLESS* packages (Evans & Murshudov, 2013 ▸; Winter *et al.*, 2018 ▸). An anomalous substructure of five Ca atoms could be determined with *SHELXD* (Sheldrick, 2008 ▸). While three of the Ca atoms had close to full occupancy, the remaining two Ca atoms had estimated occupancies of around 50% each. After density modification, over 90% of the structure could be built into the resulting experimental electron-density map. However, although the structure was solvable, it could not be refined owing to ambiguous N-terminal electron density. Therefore, X-ray diffraction data from a calbindin-D28K crystal grown using crystallization condition 2 were also collected on the I03 beamline at Diamond Light Source. Data were collected at 100 K with 30% glycerol as a cryoprotectant. 1040 images were collected with an ω oscillation of 0.10° and an exposure time of 0.1 s per image at a wavelength of 0.976 Å. Diffraction-intensity data were indexed, integrated and scaled using the *autoPROC* toolbox (Table 1 ▸; Vonrhein *et al.*, 2011 ▸). Molecular replacement with the calcium SAD model yielded a phasing solution using *Phaser* (McCoy *et al.*, 2007 ▸). After cycles of model building and refinement using *Buccaneer* and the *CCP*4 suite of programs (Cowtan, 2012 ▸; Murshudov *et al.*, 2011 ▸; Emsley *et al.*, 2010 ▸), building of the final model was completed in *Coot* and the model was refined with *BUSTER* v.2.10.2.

### 2.5. SEC-SAXS   

SEC-SAXS was performed on the B21 beamline at Diamond Light Source. Calbindin-D28K samples were dialyzed for 12 h at 277 K against 1 l sample-dialysis buffer (20 m*M* Tris pH 7.8, 150 m*M* NaCl with or without 3 m*M* CaCl_2_) prior to experiments. A 45 µl sample of 11.5 mg ml^−1^ calcium-loaded calbindin-D28K or 12.2 mg ml^−1^ unloaded calbindin-D28k was injected onto a Shodex KW402 size-exclusion column pre-equilibrated with dialysis buffer and run at a flow rate of 0.16 ml min^−1^. Intensity [*I*(*q*)] data were collected as the eluate passed through the X-ray beam and were plotted against *q* = 4πsinθ/λ. The system operated with an exposure time of 3 s at 12.4 keV (1 Å) using a PILATUS 2M detector located at a distance of 4 m. Data were analysed using *ATSAS* and *SCÅTTER* (Franke *et al.*, 2017 ▸; Franke & Svergun, 2009 ▸; Kozin & Svergun, 2001 ▸). The *FoXS* web server was used to compute the theoretical scattering profile from NMR and crystal structures of calbindin-D28K for comparison with the experimental data (Schneidman-Duhovny *et al.*, 2013 ▸, 2016 ▸). No changes were made to the calbindin-D28K protein models used. However, the solvent molecules were removed from the structures. The structures were used as deposited. PDB entry 6fie has an extra three residues at the N-terminus owing to cleavage of the His tag at the 3C protease cleavage sequence. The SAXS data and analysis have been deposited in the Small Angle Scattering Biological Data Bank (Valentini *et al.*, 2015 ▸; accession codes SASDDL6 for calcium-loaded calbindin-D28K and SASDDM6 for unloaded calbindin-D28K).

## 3. Results   

### 3.1. Crystal structure of calbindin-D28K   

**Figure 1:**
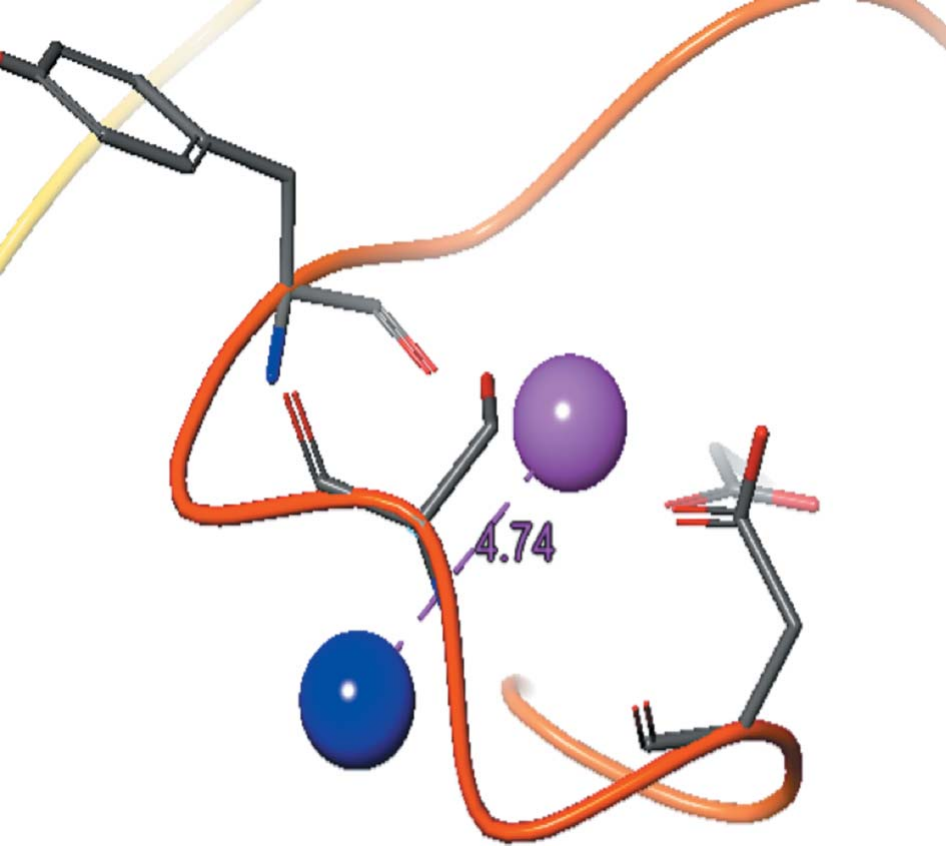
The two Ca atoms in EF1 from the substructure coordinates. The distance between the peaks is shown. There was an estimated occupancy of 50% for each calcium. The Ca atom in pink is that refined in PDB entry 6fie.

Refinement statistics for the calbindin-D28K crystal structure (PDB entry 6fie; crystal condition 2) determined to a resolution of 1.51 Å are presented in Table 1 ▸. The disorder in the first calcium-binding site is reflected in the weaker side-chain electron density in the N-terminal EF-hands, accounting for the relatively high RMSZ score for this structure compared with other X-ray structures determined at similar resolution. The calcium SAD structure determined from crystal condition 1 allowed direct visualization of the Ca atoms (Supplementary Fig. S1). This revealed for the first time that there are two conformations, with the calcium ions located 4.7 Å apart, in the N-terminal EF-hand 1 (Fig. 1 ▸). Only one of these calcium-binding conformations was visible in the electron density for both crystal conditions. Owing to the increased noise in this region, the calcium-coordinating waters were not modelled around the disordered EF-hand 1. Zhang *et al.* (2008 ▸) determined the space group to be *C*2 for a crystal obtained using crystallization condition 1. However, the crystal obtained from repeated use of this condition belonged to space group *P*2_1_2_1_2.

### 3.2. Calcium binding   

**Figure 2:**
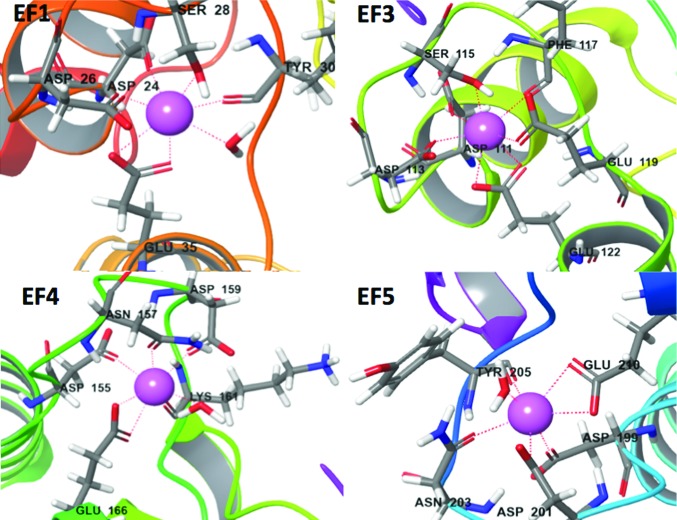
The four EF-hand calcium-binding loops of calbindin-D28K. Calcium-binding amino acids, water molecules and calcium ions are shown, while the rest of the structure is illustrated by a cartoon representation. Each calcium ion is coordinated with a pentagonal bipyramidal coordination geometry, the bonds for which are represented by a dotted line. The EF1 calcium is coordinated by Asp24, Asp26, Ser28, Tyr30, Glu35 and water, the EF3 calcium by Asp111, Asp113, Ser115, Phe117, Glu119 and Glu122, the EF4 calcium by Asp155, Asn157, Asp159, Lys161, Glu166 and water, and the EF5 calcium by Asp199, Asp201, Asn203, Tyr205, Glu210 and water.

At a Ca^2+^ concentration of 1 m*M*, calbindin-D28K binds four calcium ions at the EF1, EF3, EF4 and EF5 motifs with a pentagonal bipyramidal coordination geometry (Fig. 2 ▸). The coordinating residues, starting from the N-terminal binding residues of the calcium-binding loops, are aligned in Table 2 ▸. Five amino-acid residues, a backbone interaction and a water molecule complete the coordination of calcium in the EF1, EF4 and EF5 motifs. In contrast, the EF3 motif utilizes an interaction at Glu119 (position 9) instead of a water molecule to complete the coordination of calcium. At position 12 all four calcium-binding loops possess a conserved glutamate side chain that acts as a bidentate coordinating residue. There are also non-calcium-binding conserved residues within the EF loops; there is a conserved glycine at position 6 and either a leucine or isoleucine at position 8. There were no detectable differences in the SAXS scattering curves for calcium-loaded calbindin-D28K and the calcium-unloaded protein (Supplementary Fig. S2), indicating that the protein retains the same ‘kidney-bean’ shape on calcium binding.

### 3.3. Structural comparison   

**Figure 3:**
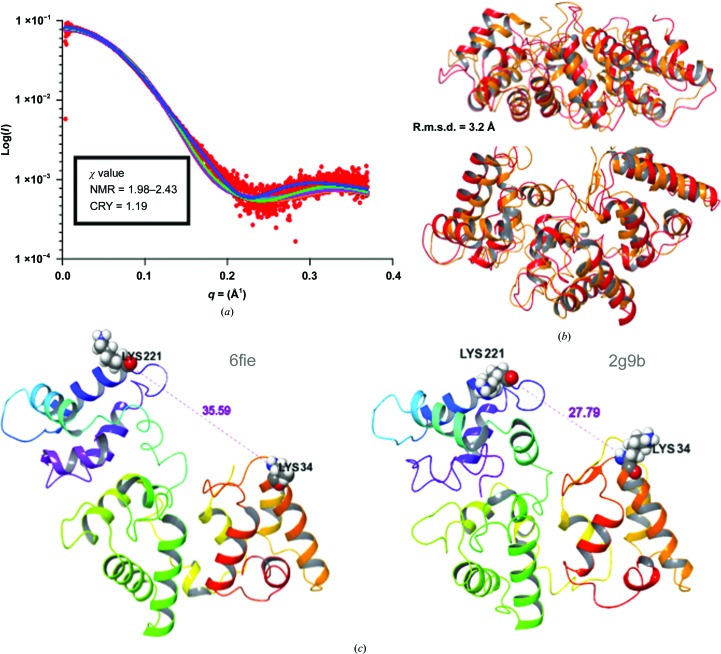
(*a*) Log_10_ SAXS intensity *versus* scattering vector *q*. The plotted range represents the positive-only data within the specified *q*-range (red). The calculated *FoXS* scattering curve of PDB entry 6fie (blue) and calculated *FoXS* scattering curves for the first five models of PDB entry 2g9b (green, turquoise, pink, orange and black). (*b*) *FATCAT* pairwise alignment and superimposition (PDB entry 6fie, red; PDB entry 2g9b, orange); there is a 90° rotation between the two images. (*c*) Measurement of the distance between the C^α^ atoms of Lys34 and Lys221; the distance is shown in Å

Calculated scattering curves for PDB entry 6fie and all ten models from PDB entry 2g9b (Kojetin *et al.*, 2006 ▸) were compared with the X-ray scattering curve of calbindin-D28K in solution. Interestingly, the crystal structure curve fitted the SAXS curve better than the NMR models, with a lower χ value of 1.19 *versus* a range of 1.98–2.43 for the NMR models (Fig. 3 ▸ *a*). Pairwise alignment with *FATCAT* (Ye & Godzik, 2003 ▸) calculated an r.m.s.d. of 3.2 Å between the crystal structure and the NMR structure (Fig. 3 ▸ *b*). The distance between pairs of C^α^ atoms in EF1 and EF6 of the calbindin models indicates that the NMR model is more compact compared with the crystal structure. For instance, the distance between Lys34 C^α^ and Lys224 C^α^ is 35.59 and 27.79 Å for PDB entries 6fie and 2g9b (model 2), respectively (Fig. 3 ▸ *c*).

## 4. Discussion   

Calbindin-D28K is a widely expressed calcium-binding protein that possesses a multiplicity of physiological functions. Its expression is particularly high in the central nervous system and absorptive epithelial tissues, where it buffers and facilitates the movement of calcium (Clemens *et al.*, 1989 ▸; Schmidt, 2012 ▸; Wasserman *et al.*, 1969 ▸). Recent work has hinted at a possible protective role of calbindin-D28K in inhibiting apoptosis and necrosis, and in slowing the pathogenesis of neurodegenerative diseases such as Alzheimer’s disease (Yuan *et al.*, 2013 ▸; Yenari *et al.*, 2001 ▸; Sun *et al.*, 2011 ▸; Kook *et al.*, 2014 ▸; Bellido *et al.*, 2000 ▸). Not only does calbindin-D28K act as a buffer for calcium ions, but it has also been shown to interact with multiple protein targets to modulate their function or catalytic activity (Shamir *et al.*, 2005 ▸; Berggård, Szczepankiewicz *et al.*, 2002 ▸; Lutz *et al.*, 2003 ▸). Here, we report the first X-ray structure of calbindin-D28K, allowing the first detailed high-resolution analysis of its calcium-binding properties. The X-ray structure of human calbindin-D28K also displays significant structural differences when compared with the previously published NMR structure of the rat calbindin-D28K molecule. The human and rat isoforms have a sequence identity of 98%. Gln44, Asp225, Thr232 and Cys257 in human calbindin-D28K are changed to Leu44, Glu225, Ser232 and Ser257 in the rat isoform. These residues are solvent-exposed and do not seem to explain the structural difference observed between the two structures. Human calbindin-D28K consists of six EF-hand motifs arranged in three pairs that maintain a globular structure stabilized by hydrophobic interactions (Kojetin *et al.*, 2006 ▸). Four EF-hands bind calcium at a concentration of ∼1 m*M* in the crystallization buffer, a concentration that is significantly higher than the usually nanomolar physiological (cytosolic) concentration. This would imply that the structure presented here represents a calcium-saturated form of the protein with respect to physiological conditions (Berridge, 1997 ▸). There are differences in the calcium-binding residues between the individual calcium-binding loops; despite these differences in primary structure, macroscopic studies have indicated that calbindin-D28K binds calcium in a nonsequential, parallel manner (Berggård, Miron *et al.*, 2002 ▸). However, other spectroscopic studies have indicated the opposite, that calcium binding is not simultaneous (Venters *et al.*, 2003 ▸), and the differences in the calcium-binding mechanisms observed in the crystal structure support this. EF3 is the only calcium-binding loop that does not use water in the pentagonal bipyramidal coordination of the calcium ion; Glu119 at position 9 instead fulfils this role. Interestingly, both EF1 and EF4 also have glutamate at position 9 of the loop, but these residues are not involved in calcium coordination. Compared with the existing NMR model of calbindin-D28K, the X-ray structure is less compact, with the intramolecular distances increased by several ångströms across the molecule. This less condensed model was validated by SAXS, as the scattering curve of the protein in solution was better predicted by the crystal structure. This is surprising as it is often assumed that crystal structures would be more compact than solution NMR structures owing to crystal-packing restraints. The high overall r.m.s.d. value of 3.2 Å also reflects significant structural differences between the X-ray structure and the NMR model. There are substantial differences in side-chain conformations both across the molecule and within the calcium-binding loops. Calcium binding at the N-terminal EF-hand (EF1) motif (where the electron density is more disordered) appears to be more flexible that in the other calcium-binding loops. The calcium SAD experiment indicated that there are two distinct calcium positions in the N-terminal EF-hand, with the calcium ions bound 4.7 Å apart in the crystal form reported by Zhang *et al.* (2008 ▸) (crystallization condition 1). Although the calcium SAD data did yield a phasing solution, the dual calcium-binding geometry precluded full structure refinement. Consequently, new crystal-growth conditions potentially favouring a single conformation were explored, yielding crystal condition 2 in which residues are visible at the N-terminus. Nevertheless, the N-terminal flexibility still manifests as structural disorder, with higher temperature-factor values in this region. It was not possible to build the two conformations into the electron density, and only one calcium-binding site is visible in the final structure. The flexibility of this region could be important in facilitating inter­actions with other proteins. Site-directed mutagenesis studies have previously revealed that inositol monophos­phatase (IMPase) binds to aspartate residues 24 and 26 in this flexible calcium-binding loop (EF1) of calbindin-D28K (Levi *et al.*, 2013 ▸). Here, we demonstrate that Asp24 and Asp26 are involved in calcium binding. It has previously been demonstrated that the potentiation of IMPase catalytic activity by calbindin-D28K occurs irrespective of calcium being present (Berggård, Szczepankiewicz *et al.*, 2002 ▸). The peptide-binding fragment of Ran-binding protein M has also been shown to induce large chemical shifts in the NMR spectrum of calbindin-D28K in the flexible N-terminal region (Lutz *et al.*, 2003 ▸). Docking simulations predict the binding of IMPase at the grooved structure between the N- and C-terminal EF-hand bundles of calbindin-D28K, where the X-ray structure differs the most from the NMR model.

The SAXS scattering curves of calcium-loaded and unloaded calbindin-D28K are essentially identical, indicating similar globular structures for both states and no increase in flexibility. Because SAXS is a low-resolution technique that can only be used to monitor large conformation changes, these findings do not preclude significant changes on the intra­molecular scale.

## 5. Conclusion   

We present here the first X-ray structure of calbindin-D28K at near-atomic resolution. Elucidation of the calcium coordination geometry in the EF-hand loops is consistent with previous reports that the protein has four calcium-binding sites. Calcium SAD at long wavelength demonstrated that the N-terminal EF-hands are particularly flexible and possesses two calcium-binding conformations. Residues that have previously been shown to be involved in protein–protein interactions are demonstrated to also coordinate calcium, potentially bestowing a calcium-sensor function on calbindin-D28K. Calbindin-D28K maintains the same globular shape in both the calcium-loaded and unloaded forms. There is also no significant increase in the flexibility of the calbindin-D28K protein in the unloaded form. Intriguingly, PDB entry 6fie is a better model of calbindin-D28K in solution, as the X-ray scattering curve produced by the protein is better predicted by the crystal structure than by the previously published NMR model (PDB entry 2g9b). We surmise that the high-resolution X-ray structure of calbindin-D28K presented here should be used as the model of choice for future experimentation and *in silico* modelling.

## 6. Related literature   

The following reference is cited in the Supporting information for this article: Thorn & Sheldrick (2011 ▸).

## Supplementary Material

PDB reference: human calbindin, 6fie

Supplementary Figures.. DOI: 10.1107/S2059798318011610/jc5014sup1.pdf

## Figures and Tables

**Table 1:** Crystallographic statistics and merging statistics produced by *autoPROC* for PDB entry 6fie (crystal condition 2) and *AIMLESS* for the calcium SAD data set (crystallization condition 1) Values in parentheses are for the highest shell.

Data set	PDB entry 6fie	Calcium SAD
Beamline	I03, DLS	I23, DLS
Unit-cell parameters		
*a* (Å)	84.62	88.09
*b* (Å)	104.22	100.17
*c* (Å)	29.61	30.69
α (°)	90	90
β (°)	90	90
γ (°)	90	90
Space group	*P*2_1_2_1_2	*P*2_1_2_1_2
Wavelength (Å)	0.976	3.02
Resolution range (Å)	29.61–1.51 (1.54–1.51)	50.9–1.98 (2.03–1.98)
No. of observations	151983 (7621)	119199 (3772)
No. of unique observations	41505 (2048)	15168 (848)
Completeness (%)	99.1 (99.8)	99.7 (60.2)
Multiplicity	3.7 (3.7)	7.9 (4.4)
Anomalous completeness (%)	91.6 (93.2)	73.8 (57.0)
Anomalous multiplicity	2.0 (2.0)	4.1 (2.3)
*R*_merge_ (%)	0.041 (0.852)	0.090 (0.970)
*R*_p.i.m._(*I*) (%)	0.024 (0.506)	0.030 (0.330)
CC_1/2_	0.998 (0.365)	0.997 (0.743)
〈*I*/σ(*I*)〉	14.4 (1.4)	13.8 (1.8)
Refinement	*BUSTER* v.2.10.2	
Resolution range (Å)	21.90–1.51	
*R*_cryst_	0.202	
*R*_free_	0.236	
No. of protein atoms	4127	
No. of ligand atoms	4	
No. of solvent atoms	180	
Mean *B* factor (Å^2^)	37	
R.m.s.d., bond lengths (Å)	0.01	
R.m.s.d., bond angles (°)	1.02	

**Table 2:** The aligned calcium-binding loops from calbindin-D28K starting from the first calcium-binding residue Calcium-binding residues are highlighted in bold. A backbone carbonyl group binds the calcium at position 7.

	Amino-acid position starting from the first calcium-binding residue												
EF-hand	1	2	3	4	5	6	7	8	9	10	11	12	Water
EF1	**Asp**	Ala	**Asp**	Gly	**Ser**	Gly	**Tyr**	Leu	Glu	Gly	Lys	**Glu**	**Y**
EF3	**Asp**	Thr	**Asp**	His	**Ser**	Gly	**Phe**	Ile	**Glu**	Thr	Glu	**Glu**	N
EF4	**Asp**	Ser	**Asn**	Asn	**Asp**	Gly	**Lys**	Leu	Glu	Leu	Thr	**Glu**	**Y**
EF5	**Asp**	Gln	**Asp**	Gly	**Asn**	Gly	**Tyr**	Ile	Asp	Glu	Asn	**Glu**	**Y**
