# Supplementary material for: The X-ray structure of human calbindin-D28K: an improved model
Source: Acta Crystallogr D Struct Biol. 2018 Oct 2;74(Pt 10):1008–14. doi: 10.1107/S2059798318011610 (PMC6173056; doi:10.1107/S2059798318011610)
Supplement: Supplementary file 1 [file d-74-01008-sup1.pdf]

STRUCTURAL
BIOLOGY

Volume 74 (2018)

Supporting information for article:

The X-ray structure of human calbindin-D28K: an improved model

James W. Noble, Rehab Almalki, Stephen M. Roe, Armin Wagner, Ramona Duman and John R. Atack

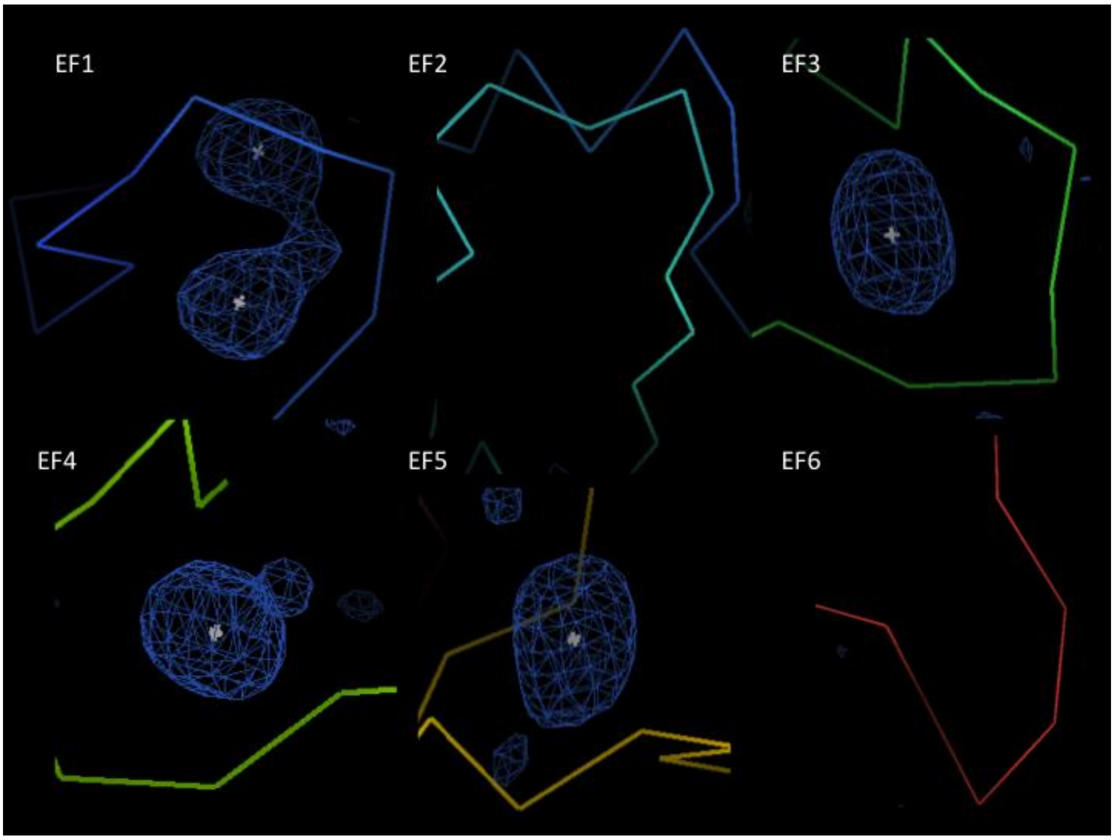

Figure S1 Anomalous Fourier map and heavy atoms computed with ANODE (Thorn & Sheldrick, 2011) . Only the calcium binding EF hands contain strong peaks in the Fourier synthesis, allowing the direct visualization of the calcium atoms from crystal condition 1. EF hand 1, 3, 4 and 5 contain calcium with EF1 having two strong peaks indicating it binding calcium in two different conformations.

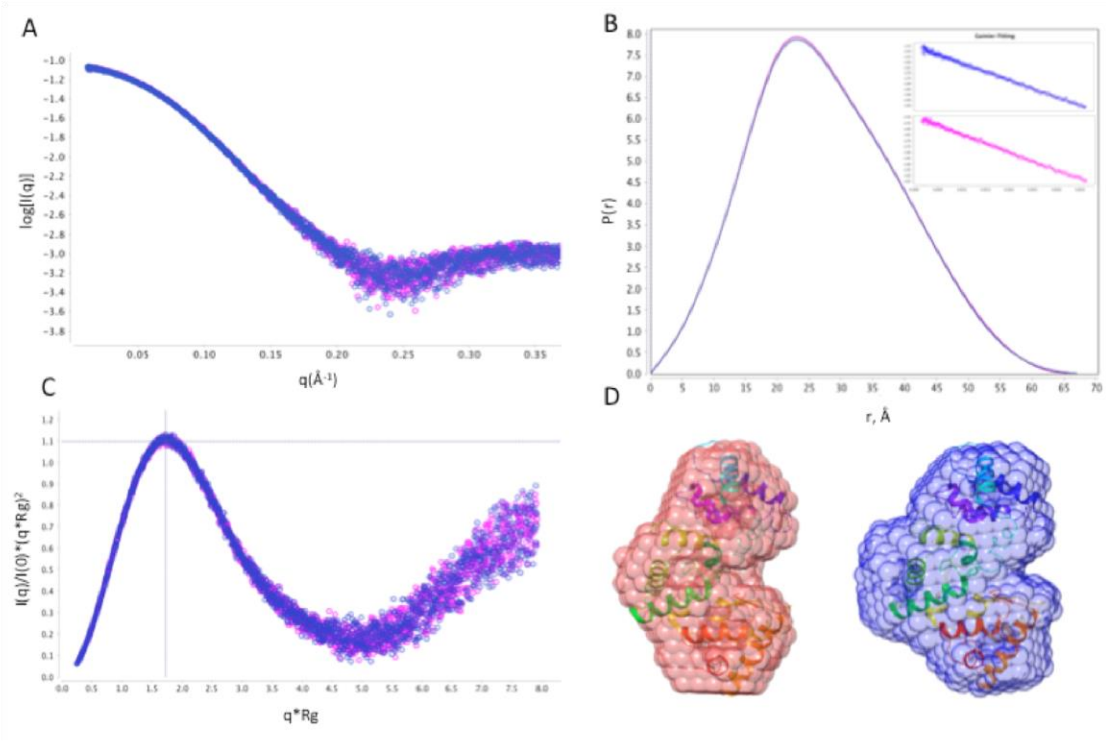

Figure S2 This figure is colour coded, blue APO and pink for calcium bound Calbindin-D28K A. Log10 SAXS intensity versus scattering vector, *q*. Plotted range represents the positive only data within the specified *q*-range. B. Pair-distance, P(r), distribution function. Maximum dimension, *dmax*, is the largest non-negative value that supports a smooth distribution function. The Guinier fitting for both data sets are also shown. C. Dimensionless Kratky plot. Cross-hair marks the Guinier-Kratky point (1.732, 1.1), the main peak position for globular particles. D. *Ab initio* envelopes with 6FIE superimposed.
